# Photonic crystals umbrella for thermal desalination: simulation study

**DOI:** 10.1038/s41598-022-24336-w

**Published:** 2022-12-13

**Authors:** Hassan Sayed, Arafa H. Aly, Thomas F. Krauss

**Affiliations:** 1 TH-PPM Group, Physics Department, Faculty of Sciences, Beni-Suef University, Beni Suef, Egypt; 2 Department of Physics, University of York, York, YO10 5DD UK

**Keywords:** Energy science and technology, Materials science, Mathematics and computing, Optics and photonics, Physics

## Abstract

For sustainable water desalination, there is a worldwide push towards solar thermal desalination with the objective to limit the amount of consumed energy in other desalination technologies and maximize the resulting freshwater from saline water. Here, we demonstrate a photonic crystals solar umbrella that covers the saline water surface, demanding to absorb all the incident electromagnetic wave and remit it as greater wavelengths in the range of mid-infrared (MIR) to be highly absorbed and localized close to the water surface. The temperature of the saline water with a refractive index of 1.3326 is reached to $45^\circ \mathrm{C}$ after one hour of illumination with the incident power intensity equal 680 $(\mathrm{w}/{\mathrm{m}}^{2})$. Hence, by adding one-dimensional PCs the surface temperature is reached $77^\circ \mathrm{C}$. Also, by adding 2D PCs to allow the vapor to flow up through the pores of the structure with the diameter of the pore equal to 500 nm, the surface temperature is reached $100^\circ \mathrm{C}$ after three hour of illumination. Thus, the effective use of electromagnetic waves and warmth localization at the surface of saline water is accomplished by radiative coupling with the effect of 2D PCs. We design the considered structure by using COMSOL multiphysics which based on the finite element method (FEM).

## Introduction

Water scarceness has become a growing global problem, affecting more than one-third of the world's population due to the lack of fresh water resources^1,2^. Thus, one of the most significant methods for dealing with water shortages is seawater desalination^3^. Desalination is the method of extracting minerals and salts from saline water in order to produce freshwater suitable for human consumption or plant irrigation^4^. Commercial desalination facilities, like newly constructed reverse osmosis (RO) plants and legacy installations with multistage flash evaporation and multi-effect distillation (MED), have partially alleviated water scarcity stress in the last decade ^5–9^. Moreover, these methods have different efficiency benchmarks for seawater and brackish water. Water with a salinity of less than 500 parts per million is usually considered safe to drink^10^. As a result, many desalination processes have been developed in order to save energy. In terms of application, the next-generation water purification technology must be able to effectively overcome the water–energy nexus and provide the following properties, such as low-cost, easy to fabricate, and low-energy requirement. Hence, to justify the clean and abundant energy requirement, we used the electromagnetic waves from the sun that incident on the earth's surface.

In a single hour, the Earth absorbs more energy from the sun than the entire human population consumes in a year. Every hour, the sun provides 173,000 TW of energy to the earth^11^. In 2017, mankind consumed 160,000 terawatt of electricity^12^. The sunlight that is incident on the earth's surface consists of three parts, as follows: 50% near-infrared region (700–2500 nm), 43% visible light (400–700 nm), and about 7% ultraviolet (300–400 nm)^13^. Recently, we have two distinctive methods to produce fresh water with the aid of solar energy. The first procedure is the freshwater extraction from seawater or waste water (i.e., desalination or eliminating pollutants); and the second method is the gathering of freshwater from air, even on a dry surface^14,15^. For the desalination procedure, in the absorption process; the short wavelengths (ultraviolet, visible, and near infra-red) energy are necessarily changed to longer wavelengths (heat). Because of, water is transparent for the short wavelengths as its absorption coefficient for these wavelengths is equal to $1\times {10}^{-2}({\mathrm{m}}^{-1})$^16^ therefore, a large portion (~ 60%) of incident solar flux is propagating through a high depth of water and causing the raise of bulk water temperature. However, water is a very strong IR absorber owing to its high absorption coefficient $1\times {10}^{4}({\mathrm{m}}^{-1})$, thus, the electromagnetic waves are absorbed and the heat is confined near to the water surface. A new methodology for solar evaporation enhancement has recently emerged, focusing on surface heating rather than wasting energy by concentrating sunlight at the water–air interface^17,18^.

Different designs of nanomaterial absorbers for surface heating have been reported with an efficiency of about 90%^19–24^. For the literature survey, in 2014, the wide-angle, mid-infrared, broadband absorber made of isotropic dielectrics and layers of graphene was studied by Ning et al.^25^. Then, in 2018, a broadband absorber, which is based on the plasma metamaterial with triangular ring-shaped resonators, can achieve an absorption rate greater than 90% in the range of incident photons from 11.76 to 14.43 GHz Zhang et al.^26^. Also, in the same year, an electromagnetic absorber was realized based on the plasma metamaterial and lumped resistors. This absorber achieved an absorption rate greater than 90% at 1.6115–4.0798 GHz^27^. In 2020, the graphene-embedded photonic crystals (GPCs) structured by the cascade structure generated with the periodic sequence and the quasi-periodic in the terahertz regime, a unidirectional absorber with an ultra-broadband absorption bandwidth and angular stability is accomplished, the proposed composite structure has a relative absorption bandwidth that is up to 94.53%, which is significantly higher than the periodic one by Guo et al.^28^. Moreover, in 2021, Wan et al. found that, the quasi-periodic structure (QPS) has a wider frequency bandwidth than the periodic structure and a wider angular selective range for the same incident frequency of the electromagnetic wave^29^, Finally, in 2022, Lan Gao et al. design a black absorber sheet (BAS) to enhance solar steam generation (SSG) with a new class of metamaterials with two-dimensional periodicity. They selected nanostructured black silicon (B–Si), which has excellent absorptivity in the solar spectrum from UV–Vis to NIR. These structures yield an 89% conversion efficiency^30^. But these structures in high salinity water cause salt accumulation that affects negatively the optical properties of the structure over the time of the solar desalination, as it results in a low rate of water evaporation. Therefore, we are dedicated to using PCs in thermal desalination. PCs are artificial periodic multilayer structures that have a periodicity in one^31,32^, two^33–36^ and three dimensions^37^. PCs have significant optical properties and electromagnetic waves localization with respect to the considered application^38^. In particular, PCs have shown a promising response towards the control of the propagation of electromagnetic waves due to their peculiar properties. PCs could confine or allow the propagation of the incident radiation as a result of providing some stop or pass bands of frequencies^39–41^. Bands of frequencies similar to those of the incident radiation are called the photonic band gaps (PBGs)^42–44^.

The objectives of this paper are to firstly convert the shorter wavelengths to longer wavelengths in the range of infrared radiation, and the second object is the localization of the thermal energy on the saline water surface. These objectives could be satisfied by adding a solar umbrella (photo-thermal device to prevent the water from evaporation directly from the sunlight) with a PCs structure to localize the thermal energy close to the surface^45–47^. In addition, these PCs solar umbrellas must have small holes to allow the vapour to flow up and float on the top surface of the saline water^48^. Finally, PC's solar umbrella is trapping all the incident electromagnetic waves and converting them into thermal energy. Thus, PCs solar umbrella is a promising candidate for economically efficient water desalination devices.

## Modeling and simulation

Simulating radio frequency (RF) heating with COMSOL Multiphysics by transferring electromagnetic waves (EMW) into thermal energy has the following steps. Firstly, electro-magnetic losses mean an attenuation of the field as the energy is transferred to the material and is often dissipated as heat. Compute electromagnetic fields and temperature fields. Finally, a couple of partial differential equations can be solved simultaneously.

Solve Eq. (1) ^57^ for the electric field.

1 $$\nabla \times \left({\mu }_{r}^{-1}\nabla \times {E}_{r}\right)={k}_{0}^{2}\left({\varepsilon }_{r}-j\sigma /\omega {\varepsilon }_{o}\right){E}_{r}$$

Then, we compute the heat source by using Eq. (2)

2 $${Q}_{e}=0.5Re\left(J.{E}^{*}\right)+0.5Re\left(J\omega B.{H}^{*}\right)$$

Finally, we calculate the temperature by using Eq. (3)

3 $$\rho {c}_{p}\frac{\partial T}{\partial t}+\nabla .\left(-k\nabla T\right)={Q}_{e}$$

Hereby, we achieve the shifting of solar radiation to mid-IR and larger wavelengths to increase the probability of thermal energy localization near to the water surface. Therefore, the inclusive efficiency (*η*) of the system when used as an umbrella over a water surface, such as an evaporation pond, can be expressed as in Eq. (4). This efficiency is a function of the absorber efficiency $({\eta }_{1})$ , the emitter efficiency $({\eta }_{2})$, and the portion of incident electromagnetic waves that results in evaporation $({\eta }_{3})$.

4 $$\eta ={\eta }_{1}\times {\eta }_{2}\times {\eta }_{3}=\frac{{m}^{^{\prime}}{h}_{fg}}{{q}_{solar}}$$

Since, ${m}^{^{\prime}}$ is the water evaporation rate, ${h}_{fg}$ is the latent heat of evaporation, and ${q}_{solar}$ is the incident solar flux.

5 $${\eta }_{1}=\frac{{\alpha }_{s}\times {q}_{solar}-{\varepsilon }_{s}\sigma {T}_{abs}^{4}}{{q}_{solar}}$$

6 $${\eta }_{2}=\frac{F{\varepsilon }_{b}\sigma {T}_{abs}^{4}}{{\alpha }_{s}\times {q}_{solar}-{\varepsilon }_{s}\sigma {T}_{abs}^{4}}$$

7 $${\eta }_{3}=\frac{{m}^{^{\prime}}{h}_{fg}}{F{\varepsilon }_{b}\sigma {T}_{abs}^{4}}$$

For studying heating procedure, we have two types; Frequency–Transient and Frequency–Stationary as we will discuss in the following.

### Frequency–Transient

**Figure 1:**
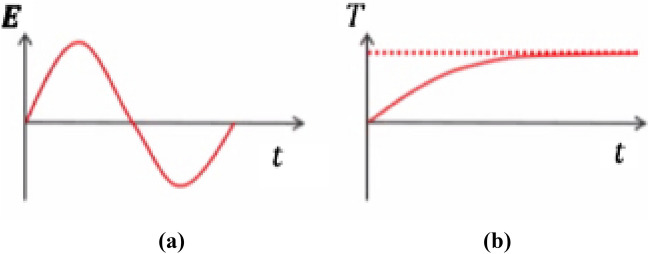
(**a**) The power dissipation as a time dependent for one cycle, (**b**) the average temperature by thermal heating as a function of time.

i. Find the cycle-averaged energy dissipations by solving the electromagnetic equation in the frequency domain as we shown in Fig. 1a
ii. Then, to discover the time-dependent temperature distribution, use this as a constant heat source in a thermal model. as we shown in Fig. 1b

### Frequency-Stationary

**Figure 2:**
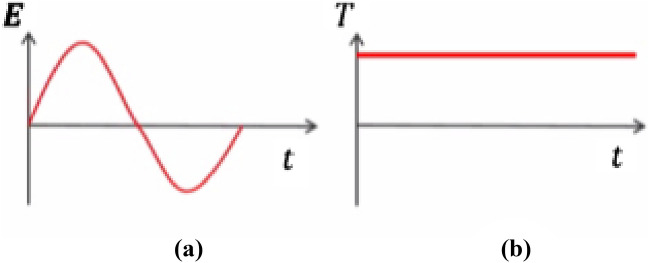
(**a**) The power dissipation as a time dependent for one cycle, (**b**) the steady state temperature by thermal heating as a function of time.

i. Solve the EM equation in the frequency domain and find the cycle- averaged power loss distribution as we shown in Fig. 2a
ii. Apply this as a constant heat source in thermal model to find the steady state temperature distribution as we shown in Fig. 2b

Finally, the solar irradiance consists of massless photons with energy. When these photons interact with PCs solar umbrella, they will be absorbed, and then they will be converted to thermal energy. Thus, the thermal energy has been calculated by the following: Firstly, by calculating the absorption power, this power is converted to kinetic energy, and this kinetic energy is considered as the thermal energy. The boundary conditions of the simulation procedure for the acrylic tank filled with saline water (which discuss in the next part) to estimate the thermal losses are: convection on all side walls equal to 5 W/m^2^ K, radiation from all side walls and water surface with emissivity equal to 0.95, and the initial temperature of water adjusted to be 20 °C. Also, the meshing size must be 10 times smaller than the smallest incident wavelength to get more accurate results in the finite element method as we discuss in our previous work at^48^. The simulation meshing parameters are the maximum element size equal to $3\times {10}^{-9} (\mathrm{m})$, minimum element size equal to $1.51\times {10}^{-10} (\mathrm{m})$, and the maximum element growth rate is 1.3.

## Results and discussions

Here, our results and discussions are displayed in two parts. Firstly, we are concerned with the thermal properties of water under the sun illumination (AM 1.5 G) and the distribution of the thermal energy through the saline water layers. Then, in the second part, we study the effect of PCs on the surface temperature of the water with the localization of thermal energy on the water surface. And finally, we conclude the results for the optimum conditions for the thermal desalination device.

### Sun illumination (AM 1.5 G) for saline water

**Figure 3:**
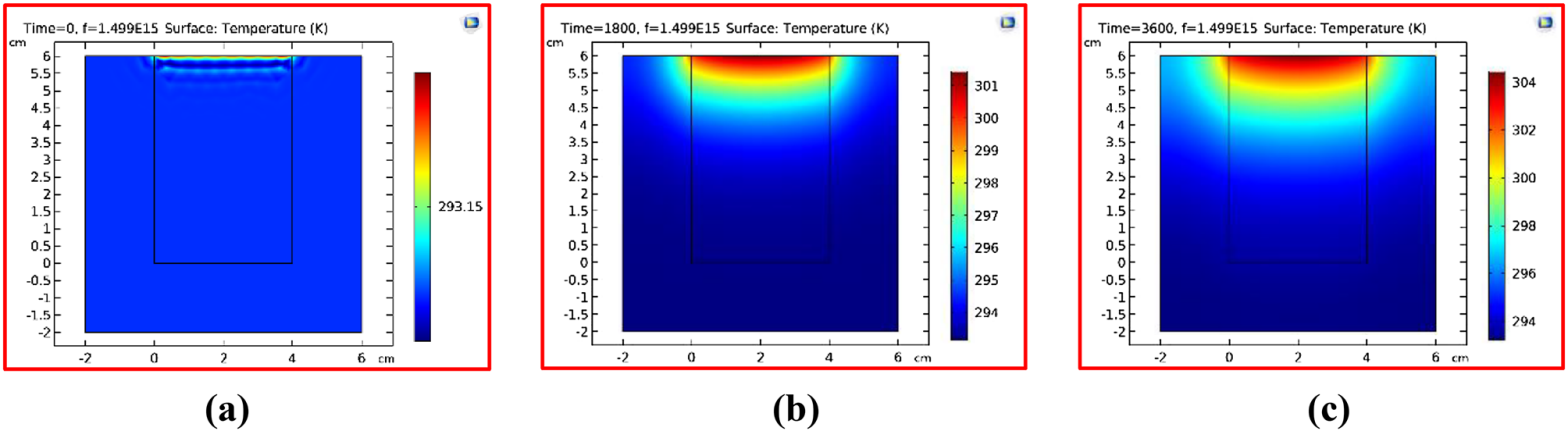
Cross-sectional temperature profile of the saline water sample surrounded by an acrylic wall on cloudy or winter days, the solar heat flux = 300 W/m^2^ at different times as (**A**) Time = 0 min (**B**) Time = 30 min (**C**) Time = 60 min.

In this subsection, our results and discussions are presented for the considered structure, which consists of saline water surrounding acrylic walls as in Fig. 3, wherein we show the results of our structure depend on the thermal properties of the used materials.

Here, in Fig. 3, we have the temperature of the saline water as it is heated with electromagnetic waves. For the Fig. 3a, at a time equal to zero minutes, the temperature of all structures is still constant at 20 C, which is the temperature of the saline water on a cloudy day in the winter season. Then, in Fig. 3b, after 30 min, we have a change in the water temperature as shown. The surface temperature reached 28 C, and also the temperature is graded from the surface to the bottom. Finally, in Fig. 3c, after one hour, the surface temperature reaches 31 C and the temperature is distributed over a large area with respect to in Fig. 3b. Also, we have the heat flux energy being distributed through a large depth of water as shown owing to the transparency of water for the visible and ultra-violet wavelengths. From this figure, we can conclude that the temperature of the water depends strongly on the time of illumination with electromagnetic waves, wherein the heat is distributed over a high depth of water. Therefore, we will work on the following shortcomings of the last structure in Fig. 3 to localize the thermal energy on the water surface to increase the evaporation rate, which affects the amount of produced freshwater and the required time for the saline water to begin the evaporation.

**Figure 4:**
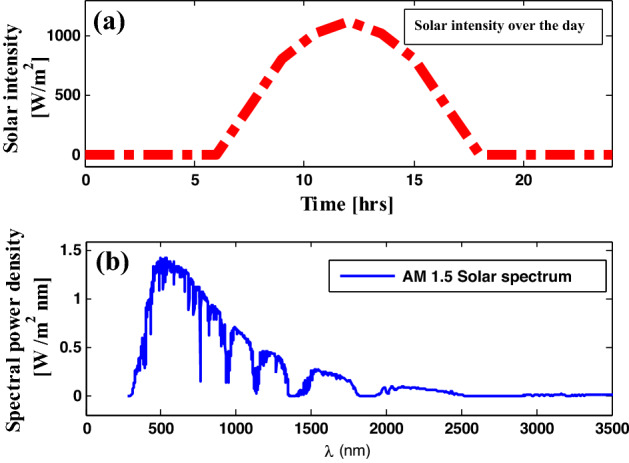
(**a**) the solar power intensity during the time of the day; (**b**) the solar spectrum AM 1.5 G, with the part of the solar spectrum that may ideally be utilized by thermal water desalination.

For the thermal water desalination device, first we determine the amount of the incident power during the day time, as in Fig. 4a. The sunrise is at 6 AM, thus the solar intensity begins to increase to reach the maximum solar power at 12 PM, and then it decreases to the sunset at 6 PM, as we have shown in Fig. 4a. The solar power is a change from 0 to $\approx 1100 (\mathrm{w}/{\mathrm{m}}^{2})$ ^49^. Despite the fact that 1367 $(\mathrm{w}/{\mathrm{m}}^{2})$ of sunlight reaches the outer atmosphere, 6% is reflected from the atmosphere, 20% is reflected from the clouds, and 4% is reflected from the surface of the earth. Thus, about 70% of the solar power is absorbed by the earth's surface ^50^. Although this power is a result of the incident solar spectrum (AM 1.5 G) as in Fig. 4b, with a high intensity of the visible light, our structure of photonic crystal solar umbrella must be a good absorber for the visible light to get high efficiency in the water desalination.

**Figure 5:**
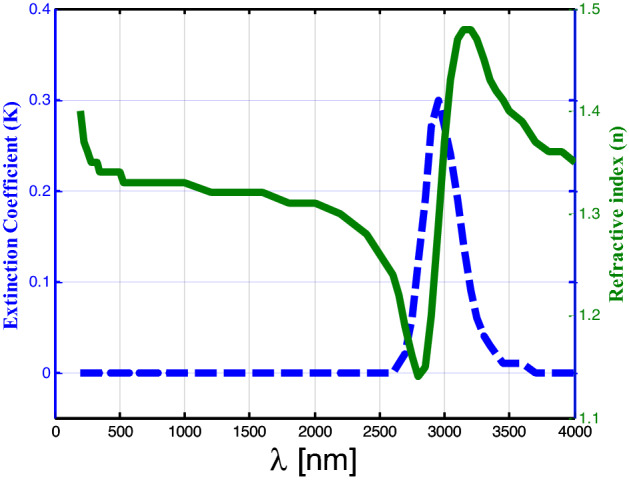
Refractive index and extinction coefficient of water as a function of incident wavelength as shown.

Here, in Fig. 5, the refractive index of water is varied from 1.15 to 1.5 RIU as a function of the incident wavelength, hence the refractive index is equal to 1.33 RIU for visible light. Moreover, as we have shown, the extension coefficient of water is varied along the x-axis of the wavelength, firstly from 200 to 2500 nm, the extension coefficient equals zero^51^, so that the water is transparent in this range of the incident wavelength, which makes sure the result of the Fig. 3. Then, from 2500 to 3000 nm, it increases rapidly and reaches 0.3. Finally, in the range from 3000 to 3500 nm, it will be decreased until it reaches zero at 3500 nm as shown. Hence, for saline water with increasing salinity and decreasing temperature, the refractive index increases as in Eq. (8) ^52,53^.

8 $$n\left(S,T,\lambda \right)=1.314+\left(1.779\times {10}^{-4}-1.05\times {10}^{-6}T+1.6\times {10}^{-8}{T}^{2}\right)S-2.02\times {10}^{-6}{T}^{2}+\left(\frac{15.868+0.01155S-0.00423T}{\lambda }\right)-\left(\frac{4382}{{\lambda }^{2}}\right)+\left(\frac{1.1455\times {10}^{-6}}{{\lambda }^{3}}\right)$$

where, S, T, $\lambda$ and n are the salinity (%), the temperature (◦C) of the seawater, the probing wavelength in nm, and the refractive index of the seawater represented in refractive index units (RIU), respectively. Thus, the refractive index of the saline water varies from 1.3326 to 1.3505 as a function of the change in salinity level from 0 to 100% at room temperature, according to the last equation.

**Figure 6:**
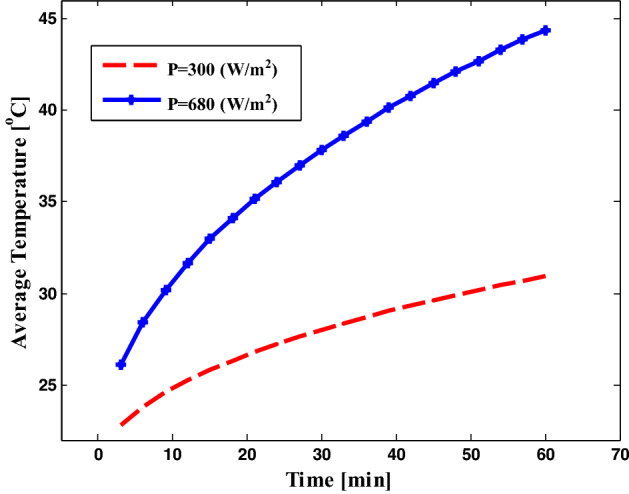
The average temperature versus time of the saline water sample surrounded by an acrylic wall on different heat fluxes as shown.

Then, by using the last results for the incident power intensity as in Fig. 4 and the dispersion relation of water as in Fig. 5, we calculate the average temperature for different incident power intensities as in Fig. 6.

Here, in Fig. 6, the resultant average temperature depends on the amount of the incident power intensity as we have shown. Therefore, the temperature of the saline water with a refractive index of 1.3326 is reached to $45^\circ \mathrm{C}$ after one hour of illumination with the incident power intensity of equal to 680 $(\mathrm{w}/{\mathrm{m}}^{2})$. Although we have a very small difference in temperature versus time curve for fresh and saline water due to the small change in refractive index between freshwater (1.33) and saline water (1.3326–1.3505), the resultant temperature is distributed over a large depth of water as we have shown in Fig. 3, we design a thermal absorber on the surface of water to localize the electromagnetic waves on the surface of water, which assists in increasing the rate of evaporation and the optimum use of solar energy. Owing to the change of incident power intensity over the day hours, the resultant average temperature changes over the day, which affects the evaporation rate.

### Photonic crystals solar umberlla

**Figure 7:**
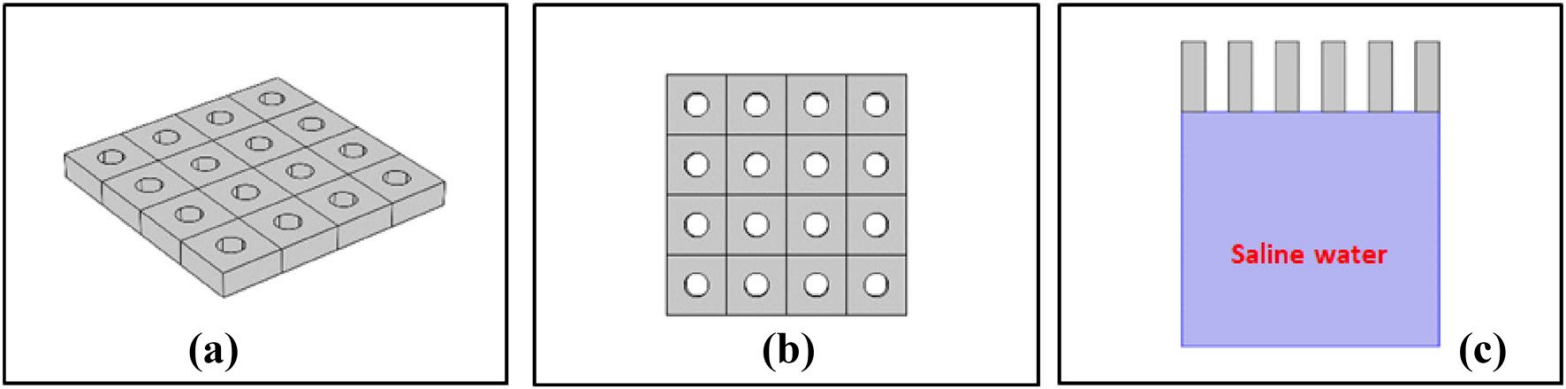
Schematic structure of 2D-PCs that act as a solar umbrella that floats on the saline water surface, (**a**) 3D structure, (**b**) upper view, and (**c**) side view with saline water as shown.

Here we integrate the unique property of the PCs about the localization of the electromagnetic waves or photons trapped in a certain area of the structure with the water desalination devices, which increase the temperature of the water surface and allow the vapor to flow up in the evaporation process. Therefore, the magic structure for this application is the two-dimensional PCs, as we show in Fig. 7. This structure floats upon the water surface, and it traps the incident electromagnetic waves. Also, the vapor flows up through the pores inside the structure.

**Figure 8:**
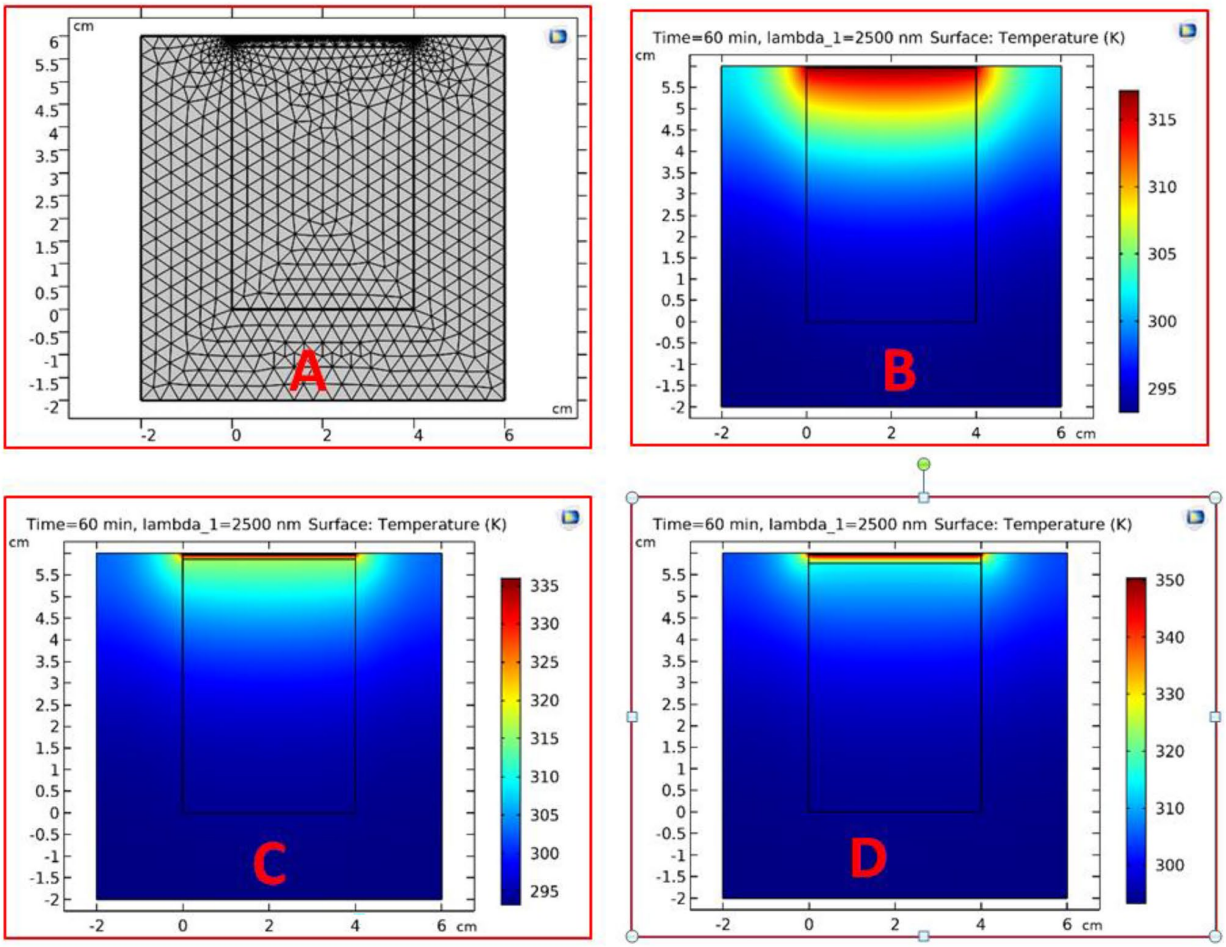
Schematic structure of saline water in an acrylic tank with a one-dimensional PCs of TiN/TiO2 with a thickness of 200 $\mathrm{\mu m}$ for each of them for one period. The solar heat flux = 680 W/m^2^ at one hour as shown (**A**) Meshing size is fine, (**B**) Without air gap, (**C**) With air gap equal to 1 mm, and (**D**) With air gap equal to 2 mm.

Here, we choose a good absorber and a good emitter material. Consequently, the designed porous structures of titanium nitrate (TiN) coated with titanium dioxide (TiO_2_) are a good candidate for the thermal desalination process due to their excellent optical and thermal properties, which have been discussed previously in the last paper^48^. TiN/TiO_2_ floats on the saline water surface also. It can absorb up to 90% of the incident solar spectrum. In Fig. 8, we have shown the thermal energy distribution for the saline water and one-dimensional PCs from TiN/TiO2 for one-period above.

**Figure 9:**
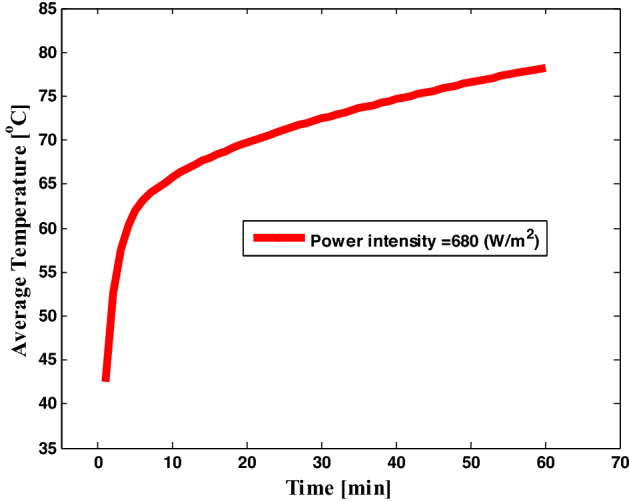
The average temperature versus time of the saline water sample with a solar umbrella as in Fig. 8 surrounding with acrylic wall.

Hence, in Fig. 8a, is the structure with a fine meshing size, because the meshing size must be 10 times smaller than the lowest wavelength to get more accurate results. We also notice that the size of the PCs layers is smaller than the water. In Fig. 8b, the temperature distribution over the structure is shown without an air gap between the saline water and the PCs structure. The temperature in this case is also distributed over a high depth of water. Therefore, we tend to insert an air gap between the water and PCs structures in Fig. 8c,d. Hence, the device validated here has the further advantage of being non-contact as in^54–56^. The air gap between the hot emitter and the calmer water surface acts as an insulting layer, so no protecting foam is needed. We have the surface temperature of saline water in the case of an air gap greater than the others, as we show in Fig. 8c,d. Therefore, in Fig. 9, we study the relation between the average temperature and the time of illumination. We find that the temperature is reached to $77^\circ \mathrm{C}$ after one hour of the incident electromagnetic waves with a power equal to 680 $(\mathrm{w}/{\mathrm{m}}^{2})$. But this structure is still inappropriate for our desalination device because it doesn't have any holes for the flow of vapor, so we devoted the research to two-dimensional photonic crystals.

**Figure 10:**
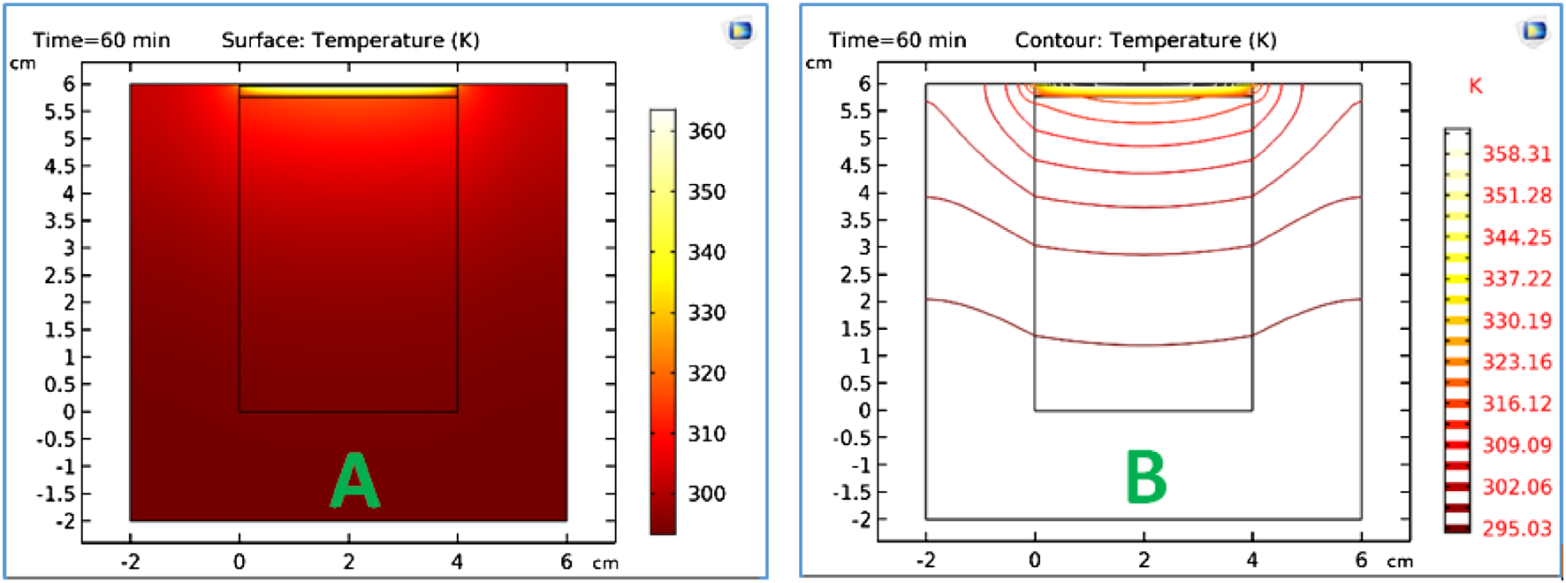
Schematic structure of saline water in an acrylic tank with a two-dimensional PCs of TiN coated with TiO2 with thicknesses donated by is 200 $\mathrm{\mu m}$ and 200 $\mathrm{\mu m}$ , respectively. The diameter of the pour equals 500 nm. The solar heat flux = 680 W/m^2^ at one hour as shown, with an air gap equal to 2 mm.

Then we replace the one-dimensional PCs above the water surface with the 2D PCs to allow the vapour to flow up through the pores of the structure as in Fig. 7c with the diameter of the pore equal to 500 nm. Here, in Fig. 10, the thermal energy distribution over the structure and the surface temperature is reached $87^\circ \mathrm{C}$.

**Figure 11:**
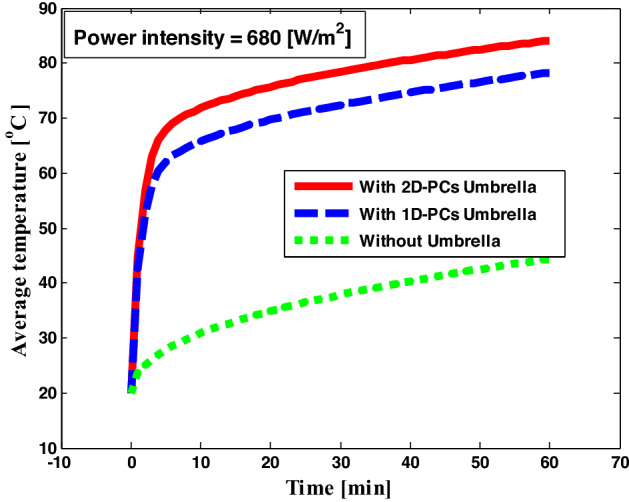
The average temperature versus time of the saline water sample with different shapes of solar umbrella.

**Figure 12:**
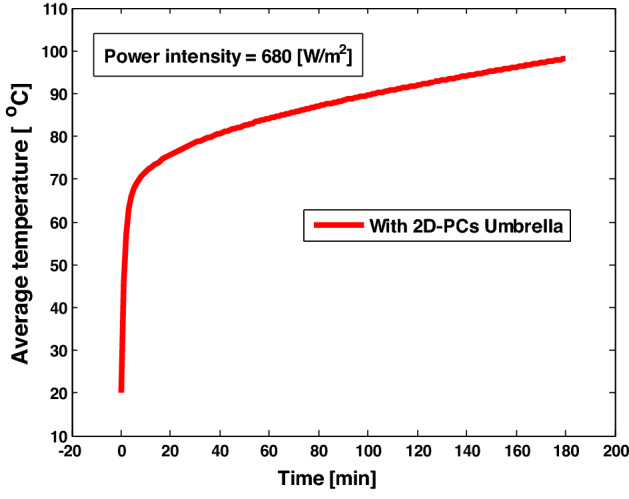
The average temperature versus time of the saline water sample with 2D- PCs solar umbrella.

Finally, we compare the different structures of our desalination device with and without PCs solar umbrella as in Fig. 11, where the average temperature in the case of 2D- PCs reaches 87 °C after one hour of the incident electromagnetic waves with the solar heat flux = 680 W/m^2^, because 2D- PCs trap incident electromagnetic waves more than 1D- PCs. Therefore, this structure assists in the evaporation mechanism and affects the water desalination efficiency. But, the resultant temperature (87 °C) is still low to evaporate the water, therefore, we increase the time of the incident electromagnetic waves on the structure to reach the degree of evaporation (100 °C) as shown in Fig. 12. In Fig. 12, the saline water temperature is reached to 100 °C after 3 h of the incident electromagnetic waves with the solar heat flux = 680 W/m^2^, and this degree of temperature is sufficient to evapourate the water. The photonic umbrella is artificially floating on the saline water surface as we adjust it to be at a small distance above the saline water surface to prevent the salt molecules from accumulating on the poures, which negatively affects the evaporation rate. The 2D-PCs structure is placed a few mm above the saline water surface to maintain a large view factor for radiation. Here, the air gap between the 2D-PCs structure and the saline water surface acts as an insulating layer, thereby eliminating the need for an insulating foam. Therefore, heat transfer in the air gap is dominated by radiation between the two surfaces due to its non-contact nature, and the convection losses are absent in the quiescent gap owing to non-contact between the two surfaces. Hence, our device here is an energy saver and the desalination process is expected to produce fresh water from saline water economically. Thus, the 2D- PCs solar umbrella is a promising candidate for water desalination technology.

## Conclusion

In this paper, we try to enhance the electromagnetic heating in the thermal desalination technology to save the energy consumed in the desalination technologies. Here we succeeded in designing a 2D-PCs solar umbrella. The designed 2D-PCs solar umbrella from titanium nitrate (TiN) coated with titanium dioxide (TiO_2_), with the diameter of the pore equal to 500 nm, achieves the following conditions for the desalination process:

1. Almost all of the incident electromagnetic waves are trapped with an absorbance of about 90%, reducing the power dissipation.
2. Convert the small wavelengths (ultra violet and visible light) to the longer wavelengths (infra-red radiation).
3. Localize the thermal energy close to the water surface to increase the evaporation rate.
4. Increase the surface temperature up to 100 °C after three hour of the incident electromagnetic waves with the solar heat flux = 680 W/m^2^.

As a result, the designed 2D-PC solar umbrella is a promising desalination technology candidate.

## Data Availability

The datasets used and/or analyzed during the current study are available from the corresponding author on reasonable request.
